# Supplementary material for: Are carbon emissions trading and green financial instruments synergistic? -Comprehensive quantitative research based on content analysis
Source: PLoS One. 2024 Mar 7;19(3):e0298601. doi: 10.1371/journal.pone.0298601 (PMC10919601; doi:10.1371/journal.pone.0298601)
Supplement: S1 Data — (ZIP) [file pone.0298601.s001.zip › Data/地级市Z_F工作报告.zip!/─┌├╔╣┼/║⌠║═║╞╠╪╩╨/└┤╫╘╣1⁄2╓┌║┼í╛┬φ┐╦╩2╛▌═°í┐_2014.docx]

各位代表：

现在，我代表市人民政府向大会报告工作，请予审议，并请各位政协委员和列席会议的同志们提出意见。

一、过去一年工作的回顾

2013年是本届政府的开局之年，也是我们积极应对复杂局面、应对各种困难挑战、取得重要发展成就的一年。在自治区党委、政府和市委的正确领导下，全市上下深入贯彻落实党的十八大精神和自治区“8337”发展思路，立足当前、着眼长远，扎实推进活力首府、美丽首府、和谐首府建设，统筹抓好稳增长、调结构、促改革、惠民生各项工作，较好地完成了全年目标任务。

(一)经济发展保持了稳中有进的良好态势

全市地区生产总值完成2710.4亿元，同比增长10%。地方财政总收入完成360.1亿元，同比增长13.8%，跃升到全区第二位;地方财政总支出292.9亿元，增长6%，其中用于民生支出达到167亿元，占地方财政总支出的57%。固定资产投资完成1504.8亿元，同比增长15.6%。社会消费品零售总额完成1142.4亿元，同比增长11.8%。城镇居民人均可支配收入完成35629元，增长9.1%;农民人均纯收入完成12736元，增长12.1%。对外贸易进出口总额实现16.2亿美元。全市经济呈现增长平稳、结构优化、质量提高的良好态势。

现代农牧业生产稳步推进。全年新开工建设蔬菜保护地3.4万亩，当年建成投产1.6万亩，启动建设8个标准化育苗中心。新开工建设大型奶牛牧场15个，加快中小型牧场整合，新增入园奶牛3.65万头，规模化养殖水平明显提升。全年完成苜蓿草种植25万亩，牧草种植专业化水平和优质饲草料供给保障能力明显提升。积极发展城郊型休闲观光农业，回民区东乌素图村、新城区水磨村等一批示范作用较强的观光新村初具规模。

工业经济实现了逆势增长。全市规模以上工业增加值增长18.1%，高于全区平均水平6.1个百分点;工业企业实现税收150.5亿元，跃居全区第二。突出抓好重点行业、重点企业达产达效，其中确保了中石油500万吨炼油项目实现稳定生产，使其对工业增加值、税收的快速增长做出突出贡献。切实加大工业运行调度力度，及时落实和制定出台自治区及我市的一系列扶持政策，光伏材料产业呈现强劲发展势头，乳业、电力、电子信息、生物医药等优势产业均实现稳定增长,全市35户工业企业产值增量过亿，并有15户企业成长为规模以上企业。高新技术产业加快发展，金山开发区升级为国家级高新技术产业开发区。

现代服务业发展呈现强劲势头。云计算产业初具规模，中国电信信息园和中国联通西北基地一期工程部分项目投入使用，百度、阿里巴巴等企业作为首批用户正式入驻，中国移动数据中心正式开工，宜齐网购、东鸽e购等本地电商企业迅速发展壮大，我市被批准为国家电子商务示范城市。制定实施物流业发展规划，开工建设了一批专业化物流基础设施项目。除我市传统的几大节庆活动外，成功举办首届云计算博览会、惠州商品展销会、首届内蒙古绿色农畜产品博览会等一批大型展会，全市会展经济呈现数量增加、规模扩大、档次提升、带动力增强的良好势头。金融业自身发展和服务地方经济发展的能力不断提升，全市新增金融机构12家，金融机构存贷款余额同比分别增长16.6%和15.3%。旅游业发展成果显著，清水河老牛湾、土左旗哈素海分别成功申报为国家地质公园、国家级湿地公园，大召区块文化产业群落创建成自治区文化产业示范基地，全年接待旅游人数、实现旅游收入同比分别增长18%和23%。

县域经济和非公有制经济加速发展。因地制宜推进县域经济发展，托克托县进入全国中小城市综合实力百强县和最具投资潜力中小城市百强县，赛罕区成为全区第四个财政收入超百亿元旗县区。出台了关于大力发展非公有制经济的实施意见，全市个体工商户和私营企业注册资金达到1126亿元，个体工商户和私营企业分别达到13.6万户和3.4万户。

健全完善了项目推进机制，开通重大项目“绿色通道”，全年组织实施各行业亿元以上项目490项，其中10亿元以上项目173项，新项目引进取得明显进展。项目建设成为拉动投资实现稳定增长的重要支撑力量。

(二)城市建设管理水平明显提升

城乡规划体系进一步完善。启动《大呼和浩特城市远景发展战略规划》编制，城乡发展、产业发展空间布局更加合理。完成城市东部新区概念性规划编制和沙尔沁工业新区总体规划编制，着力加快沙尔沁工业新区建设。中心城区实施小黑河两岸部分地段、火车站周边、如意总部基地等城市重点区块景观设计、交通组织规划，完成城市便民设施、中小学幼儿园等社会民生领域规划编制论证。出台小城镇规划建设技术导则，对一批重点城镇进行了规划修编。

城市基础服务保障能力再上新台阶。全年组织实施市政道路桥梁工程114项，改造小街巷55条，启动了南二环快速路、北出城口立交桥、中山路地下通道等一批重点工程，兴安路、昭君路等重点路段跨小黑河桥建成通车。加大雨水排放、污水处理和热源厂建设力度，生活污水集中处理率、集中供热普及率分别提高到95%和94.1%，中水回用率达到30%，对76处城市主要积水点和186个小区的供水二次加压设备进行了改造治理，新铺设地下各类管网186公里。启动实施“气化呼和浩特”工程，对64座燃煤锅炉房进行了燃气化改造及拆并整合。大力发展公共交通，新增新能源公交车430台，将免费乘坐公交车老年人范围由70岁扩大到60岁以上，在城市核心区开通了“青城1号”和“青城2号”2条全免费公交线路，投放城市公共自行车5100辆。

城市管理水平显著提升。以构建强力高效的“大城管”格局为目标，从体制机制上对加强城市综合执法改革、完善城市管理进行了有效探索。组建成立环卫产业发展有限公司。加强城市环卫基础设施建设，道路机扫、洗扫覆盖率超过65%。开工建设便民市场100个，将城郊接合部、出入城口、新建道路等区域纳入城市管理范围。基本完成数字化城管平台建设。

各类房地产建设市场保持平稳。完成城中村和棚户区、出城口拆迁改造面积863万平方米，开工建设回迁房216.2万平方米，开工建设各类保障性住房20393套，开工率达到132%，玉泉区康居家园、赛罕区大台村回迁安置房交付使用，对350个老旧小区实施了整治改造。切实加强房地产市场监管，合理控制土地供应及开发规模，全市房地产市场保持平稳发展态势。

(三)城乡发展的基础保障能力显著增强

交通、电力等基础设施建设取得重要进展。准兴运煤重载高速、京藏高速呼包段改扩建工程、呼杀高速、呼塔一级公路建成通车，国道109线十七沟至大饭铺高速、京新高速呼市段主体完工，武川至葛根塔拉一级公路、省道210线和林城关镇改线工程、清水河老牛湾旅游公路、托县沿黄公路开工建设。实施城市电网建设项目26项，完成农村电网升级改造工程。白塔机场迁建计划通过国家发改委、民航总局专家论证。编制完成城市轨道交通建设规划。

重点生态工程扎实推进。全年完成人工造林17.7万亩，封山育林17.3万亩。扎实推进北部大青山前坡生态保护治理工程，累计实现绿化面积6.2万亩，区域内23个城中村综合改造工程进展顺利，建设了新城区万亩城市草原，新建乌素图公园、哈拉沁生态保护区、植物园等4个大型公园，我市被命名为自治区级园林城市。启动大黑河河道治理工程，大型灌区节水改造、病险水库除险加固、中小河流治理、水土保持治理各项工程快速推进，全年解决近4万人安全饮水问题。

资源及环境保护得到加强。在全市范围内组织开展环保执法、打击“两违”用地等专项行动，立案查处79家环保不达标企业、966宗违法用地行为，对8家制药企业实施关停整改处理，全面清理了城区及公路沿线的煤炭物流市场。主要污染物实现达标排放，其中二氧化硫和氮氧化物提前完成“十二五”减排目标，全市环境空气质量在北方15个省会城市中始终居于上游水平。

(四)社会事业民生保障工作深入民心

就业和社会保障工作取得明显成效。全年开发公益性岗位1100个，城镇新增就业超过4万人，城镇登记失业率控制在3.8%以内。城乡低收入群众收入水平进一步提高，市四区、五个旗县城市低保标准分别提高到每人每月550元和500元，农村低保标准提高到每人每年3500元，农村五保集中供养和分散供养标准分别提高到每人每年7500元和4800元，新农合人均筹资标准提高到364元，困难家庭大学生救助标准达到每人6000元。制定出台原国有集体企业大龄下岗失业人员社会保险缴费救助意见，6700余人的保费缴纳困难问题得到妥善解决。为全市环卫工人提高工资10%，并全部办理了大额意外伤害保险。启动39个新农村示范村和28个整村推进扶贫开发项目，全年减少贫困人口1.9万人。认真落实自治区惠农政策，为中低收入农户每户发放燃煤补贴600元。

各项社会事业全面进步。坚持教育优先发展，全年启动新建、改扩建56所幼儿园，实施内蒙古大学附属中学和呼和浩特职业学院新校区、第十七中学扩建等一批重点项目。医疗卫生事业加快发展，市第一医院老年病房楼即将投入使用，中蒙医院、妇幼保健院项目主体完工，启用9个新建社区卫生服务中心。市儿童福利院、流浪未成年人救助保护中心投入使用，启动市级示范老年公寓、残疾人综合服务中心建设，累计建成50个老年人日间照料中心、8个农村互助养老幸福院。文化广电事业进一步发展，举办了首届皮画艺术品展览会，大型二人台现代戏《花落花开》荣获全国舞台艺术精品工程奖和文化部文华剧目创作奖，大型民族舞台剧《马可·波罗传奇》在美国完成百余场商业演出，全市开展800余场文化进社区活动，被文化部授予群星奖。内蒙古旧博物馆和国际会展中心改建工程顺利完工，市传媒大厦正式投入使用，62个乡镇综合文化站全部建成，全面完成“村村通”广播电视工程。启动实施市游泳馆、体育学校等一批重点项目，群众性体育活动蓬勃开展。全面启动全国文明城市创建活动，城市文明水平显著提升。

社会管理得到明显加强。社区网格化管理不断深化，广泛梳理收集各类矛盾问题，化解率达到90%以上。全市90%的社区用房面积达到300平方米以上，为每个社区新增设2名治安管理协警，城市管理志愿者队伍不断壮大。集中力量解决了一批重点领域的信访问题，全市信访总量下降32.1%。扎实推进平安创建工程，开展重点领域专项整治，严厉打击各类违法犯罪活动，发案量大幅下降，破案率明显上升，维护了首府稳定大局。强化安全生产专项检查，全市各类安全事故同比下降9.8%。

人口和计划生育工作不断加强，当年人口出生率控制在8.14‰。民族、宗教、人防、气象、防汛、防震减灾、侨务、外事、妇女儿童、老龄、残疾人、关心下一代、红十字、慈善、史志、档案等各项工作都取得了新成绩。积极支持国防和军队现代化建设，注重军民融合式发展，国防动员、民兵应急力量建设取得新成绩，优抚安置工作取得新进展。

(五)深化改革扩大开放取得新成果

深入推进政府机构改革。除实施“大城管综合执法”改革外，围绕加强食品卫生监管能力建设，与自治区质监局、食药监管局合作共建了“中国呼和浩特国家级乳肉制品检测中心”。完成市文化局、广播电影电视局、新闻出版局“三局合一”，推动新组建的民族演艺集团逐步走向市场。加快国有企业改革步伐，对春华水务等5家企业实行政府直接管理，试行国有企业职业经理人制度。完成白塔空港物流园区和出口加工区合并，规划建设新的综合保税区。

不断加大招商引资力度。精心组织呼和浩特投资贸易洽谈会、中华冀商草原行、中法企业项目对接会等大型招商引资活动。组团参加了蒙粤科技合作活动周、蒙港澳经贸合作周活动和广交会、西洽会等大型展会，在更高层面、更广范围与国内外大型企业建立起了合作联系。全年共实施国内外招商引资项目470项，实际到位国内资金678.4亿元、国外资金8.83亿美元。

全面提升对外开放水平。先后承办了环渤海区域合作市长联席会第16次市长会议和呼包银榆经济区首届市长联席会议，成功举办了第七届中国民族商品交易会、第十四届呼和浩特昭君文化节和第三届中国少数民族文化旅游艺术节，与广州市、三门峡市缔结为友好城市。

(六)政府自身建设得到新加强

加强政府班子建设。坚持中心组学习制度，认真学习贯彻党的十八大精神、习近平总书记系列重要讲话精神和自治区“8337”发展思路，结合实际谋划和加快首府发展，推动首府科学发展的能力不断提高。弘扬求真务实的工作作风，遵循客观规律办事，努力做到决定的事项说了不忘、盯住不放，狠抓落实，政府班子的执行力有了新的提高。认真贯彻民主集中制原则，完善了政府工作规则和决策程序，政府领导班子及全体组成人员讲团结、重大局，齐心协力干事创业，班子的凝聚力和战斗力有了新的提高。

认真贯彻落实中央八项规定、自治区28项和我市30条配套规定，严格按照要求组织开展了公务用车、办公用房清理治理工作，走在了全区前列。全年“三公”经费支出压减30%，市本级公务接待、公务用车运行维护及购置、因公出国(境)费用支出分别减少48.5%、75.4%、53.2%。在全市范围内深入开展“机关作风建设年”活动，着力解决“四风”方面的突出问题，行政机关和干部作风明显好转，行政效能不断提高。

大力推进政务公开，着力深化行政审批制度改革，全面提升三级公共服务平台标准化建设水平，三级政务大厅受理事项办结率达100%。全面巩固和深化公共资源交易改革，进一步完善公共资源交易市场管理制度和工作流程，完成进场交易项目919项，交易总额524亿元。充分发挥审计、监察部门的作用，加大了对保障民生领域、重点项目资金使用、国有企业的审计力度，加强和改进行政监察工作。

认真执行市人大及其常委会决定，自觉接受政协民主监督，广泛听取各民主党派、工商联和无党派人士意见，全年共办理自治区和我市两级人大代表议案、建议和政协委员提案818件，办复率均为100%。扎实开展“六五”普法工作，深入推进依法行政。修订完善地方性法规3部，制定修改政府规章2件，审结行政复议案件51件。

各位代表!过去一年的成绩来之不易。这是自治区党委、政府和市委正确领导的结果，是全市各族人民团结奋斗、顽强拼搏的结果，也是社会各界大力支持和帮助的结果。在此，我代表市人民政府，向辛勤工作的全市各族干部群众，向人大代表、政协委员，各民主党派、人民团体，向所有关心支持呼和浩特建设与发展的同志们、朋友们，表示崇高的敬意和衷心的感谢!

在看到成绩的同时，我们也清醒认识到面临的困难和存在的问题。一是经济下行压力较大。受大环境影响，部分行业和企业市场需求低迷，而且，从现有的基础支撑看，我市已实现了较大的经济规模，继续保持快速增长的难度增大。二是产业转型升级步伐还不够快。现代农业建设投入不足，部分设施农业建设成果未能充分得到利用;工业“短板”仍然明显;服务业特别是现代服务业发展滞后，首府综合优势效应发挥不够充分。三是随着城市人口的快速增长，城市基础设施建设承载力不够、精细化管理不到位等问题依然突出。四是城乡发展不协调，农村基础设施不完善、公共服务水平不高，推进城乡一体化发展任务十分艰巨繁重。五是群众关心的就业、教育、医疗、出行、食品安全、环境保护等方面存在的问题还没有妥善解决。六是政府自身建设有待进一步加强，推动和落实科学发展的能力需要进一步提高，深化改革、扩大开放的力度需要进一步加大，“四风”问题还不同程度的存在。对此，我们要采取有力措施，认真加以解决。

二、今年的主要工作任务

2014年是深入贯彻落实党的十八届三中全会精神、全面深化改革的开局之年，也是加快转型发展、实现“十二五”奋斗目标的关键一年。做好今年的工作，意义重大。

今年政府工作的总体要求是：深入贯彻落实党的十八届三中全会、中央经济工作会议精神及习近平总书记视察内蒙古重要讲话精神、自治区“8337”发展思路、自治区党委九届十次全委会暨全区经济工作会议精神和市委十一届五次全委会暨全市经济工作会议精神，以开展第二批党的群众路线教育实践活动为推动力，坚持稳中求进的工作总基调，把改革创新贯穿于经济社会发展各个领域各个环节，立足于稳，着眼于进，更加注重转型发展，更加注重结构调整和产业升级，更加注重经济增长的质量和效益，更加注重生态环保和民生改善，努力推动经济平稳持续健康发展、社会和谐稳定。

2014年主要预期目标为：地区生产总值增长9%;规模以上工业增加值增长12%;公共财政预算收入增长8%;固定资产投资增长15%;社会消费品零售总额增长11%;城镇居民人均可支配收入增长9%，农村居民人均纯收入增长10%;城镇登记失业率控制在3.8%以内。

提出上述目标，从我们所面临的困难和压力看：今年经济形势依然复杂多变，我国经济已由高速增长转入中高速增长阶段，增长速度换挡期、结构调整阵痛期、前期刺激政策消化期“三期”叠加，不确定不稳定因素增多。全区经济由于受市场需求不足，特别是能源、资源需求不足的影响，增长速度也开始调低。综合各种因素，我市适当放缓增速，符合当前的实际，有利于缓解资源、资金、市场等各种关系，更有利于为转方式、调结构、深化改革创造条件。从我们面临的机遇和动力看：我市发展已经站在新的起点，基本面向好，我们正处于转型发展的战略机遇期，处于深化改革的重要窗口期，处于新型工业化、信息化、城镇化、农业现代化的加速发展期，自治区对首府建设发展的高度重视，为我们加快发展创造了不少有利条件和积极因素。只要我们牢牢把握住稳中求进这一总基调，加快转型升级步伐，呼和浩特必将实现新一轮更高质量的发展。

完成好以上目标，今年要重点抓好以下八个方面的工作：

(一)强化工作措施，实现“三农”工作新突破

全面落实中央一号文件及农村工作会议精神，改善农村发展条件，夯实农村发展基础，深化改革，建立健全加快农村发展、城乡一体化发展的体制机制。

落实全市城乡发展空间布局规划，加强城乡一体化发展的交通基础设施保障能力。完善旗县城镇与中心城市、与工业园区及重点旅游区之间的交通网络，重点推进呼武公路另一幅、武川至葛根塔拉一级公路、呼塔一级公路二期、省道210线和林县城关镇改线工程、托县沿黄公路、清水河老牛湾旅游公路等项目建设，组织实施好国家、自治区在我市境内的重点交通工程。

修编完善各旗县城镇建设总体规划，加快城关镇和重要节点镇的建设。加强旗县城镇道路、供排水、供热、垃圾处理等各类公用设施建设，推进各旗县污水处理厂专业化运营。推动旗县城镇棚户区改造，因地制宜打造各具特色的中小城镇，增强城关镇、建制镇的集聚力和辐射带动力。

完善政策措施，落实好各项生产任务，继续加快现代农牧业发展。今年设施蔬菜基地建设的重点是要通过支持各类企业参与、引进科技人才和能手、创新大学生就业渠道、加大对本地农民及务工人员培训、提供金融服务等办法，全面提高近两年设施农业建设成果的投产应用率，同时继续加大对投产比率高、效益好的地区扩大建设面积的支持力度。继续加快推进奶牛规模化养殖进度，提升发展水平，完成176个中小牧场和119个养殖小区整合工作，新增奶牛10万头，年内在全市范围内全部实现奶牛规模化养殖。大力发展优质牧草种植业，集中连片建设一批千亩、万亩种植区，新增种植面积10万亩。巩固提高马铃薯、食用菌、肉羊产业，新建10万亩马铃薯种薯原种繁育基地、5000亩食用菌生产基地，争取肉羊出栏突破300万只。提高农田标准，稳定粮食产量。推进现代农业示范园区建设，重点打造土左旗10万亩现代农业示范园区。

分类推进新农村建设。坚持以农民为主、政府引导的原则，今年要沿重要交通干线集中连片建设56个示范村，改善村容村貌，保留村庄特色，形成示范效应。坚持把公共服务建设的重点放在农村，争取在全区率先实现农村危房改造、安全饮水、街巷硬化、村村通电、村村通广播电视和通讯、校舍建设及安全改造、标准化卫生室、文化活动室、便民连锁超市、农村常住人口养老医疗低保等社会保障“十个全覆盖”，加快促进基础设施向农村延伸、公共服务向农村覆盖、城市文明向农村辐射。

创新扶贫开发模式。以减少贫困人口为目标，实行分类指导。对符合一定条件的贫困人口，积极探索扶贫和农村低保、扶贫和养老并轨等新模式，提高扶贫开发工作的针对性和实效性，实现精准化扶贫。充分尊重农民意愿，因地制宜、合理确定移民搬迁规划。坚持领导包扶制度，启动新一轮30个整村推进扶贫开发项目。加大对贫困家庭子女上学和就业的扶持力度，对升入大学的学生由现行的一次补助改为在校学习期间给予全程补助，并在毕业后给予就业扶持政策。对农村青壮年劳动力政府提供免费职业技能培训。年内完成2万贫困人口脱贫任务。

加快生态建设步伐。抓好国家天然林保护工程、退耕还林工程、京津风沙源治理工程，完成人工造林17.7万亩，封山育林17.3万亩。实施好重点区域绿化工程，对全市主要公路、铁路交通干线两侧进行纵深绿化，加快推进农业节水灌溉、小流域治理等水利工程建设。

全面深化农村综合改革。创新农业经营主体，发展专业大户、家庭农场、农民合作社、农业企业为主的多种经营模式，在农民自愿前提下，加快推进土地流转，促进农业规模化发展。高度重视完善农业技术推广体系建设，强化农业科技成果转化应用，建立务实有效的农村科技人才培养引进机制。推动农村产权制度改革，赋予农民更多财产权利，加快建立新型工农城乡关系。创新农业投入模式，组建农业投资公司，整合财政支农资金，实行市场化运作、企业化管理，放大资金投入效应。加快推进户籍制度改革，全面放开我市市域范围内农村户籍人口进城、城镇人口在中心城区与小城镇之间、小城镇与小城镇之间的落户转移，推进农业转移人口市民化、城镇人口规模化。积极鼓励社会资本投向农村，允许各类企业和社会组织在农村兴办养老、教育等社会事业，加快提升农村公共服务水平。

(二)推动产业优化升级，增强工业经济活力

坚持做大工业总量与提升产业层次并重、与提高质量与效益并重的原则，通过“培育一批、扶持一批、引入一批、淘汰一批”，增强工业经济活力，推动工业经济在转型升级中加快发展。

培育打造一批具有一定规模和比较优势的产业集群。推动伊利、蒙牛向世界乳业10强迈进，支持蒙羊肉业争创全国知名绿色品牌，扶持可口可乐、康师傅、燕京、利乐、统一、娃哈哈等企业发展壮大，把绿色食品产业打造成为首个营业收入超千亿元产业集群。加快和林电厂、大唐托电五期、抽水蓄能电站、京能盛乐电厂以及一批重点用能企业自备电厂建设，大力发展风电、光伏发电，新增电力装机250万千瓦以上，进一步壮大以电力为核心的清洁能源产业集群。用足用好产业支持政策，扶持神舟硅业、天津中环等企业做大做强，推动华夏聚光一期、晶环蓝宝石、晟纳吉一期加快建设，积极引入晶龙集团、瑞诺机械制造和晶体生长、海霏光伏、杭州赛昂等光伏项目，推动光伏产业全产业链延伸、集群化发展。高起点、高标准发展现代化工产业，加快推动中石油千万吨级炼油基地建设。充分发挥水煤组合优势，推动庆华煤基清洁能源多联产、内蒙古矿业40亿立方米煤制气等项目早日开工建设，打造现代煤化工示范基地。

扶持发展一批具有较强发展潜力的高新技术企业。高起点编制实施金山国家级高新技术开发区发展规划，加快把金山国家级高新技术开发区建成全市高新技术产业发展高地。整合金川、鸿盛等园区科技创新资源，出台优惠政策，设立专项发展基金，引导创新要素向这几个园区集聚，促进科技成果加快转化，全面提升现有产业，集中发展高新技术产业、创新型产业。大力支持天一环境、F12高强有机纤维、银宏干细胞、浩源碳纤维、精诚绝缘子等具有明显技术创新优势的企业发展壮大，年内再培育发展20户高新技术企业。

引进承接一批具有一定竞争优势的产业转移项目。确保TCL和创维各300万台液晶电视、北京东亚铝业、广银铝业等已转移项目尽快达产达效，加快实施杭州锦江集团煤电灰铝一体化、中国北车集团西北制造基地、天登通用航空制造、白俄罗斯大型农机装备制造等项目，打造新的优势产业集群。积极主动融入环渤海经济圈，加强与粤港澳、长三角区域的交流合作，继续承接引进一批发达地区产业转移项目，努力把土地、电价、区位优势转化为吸引投资、推动发展的现实生产力。

淘汰一批产能过剩、环境污染严重、缺乏竞争优势的企业和项目。充分发挥市场机制作用，综合运用法律、经济等手段，加快形成有利于落后产能退出的机制。引导强化企业环保意识，加大环保节能改造力度，坚决淘汰环保不过关的企业和项目。强化节能节地节水、环境、技术等市场准入标准，建立健全防范和化解产能过剩的长效机制。

围绕项目入园和提升园区承载力，加大工业投资力度。深入落实自治区沿黄沿线产业带发展规划，加快推进工业集中区建设，工业投资力争超过300亿元。围绕产业转型和项目投产达产抓好工业经济运行，提高工业经济对全市经济发展的贡献率。

(三)发挥首府优势，加快发展新型现代服务业

服务业是我市经济发展的稳定器和加速器，也是发挥首府效应的重点产业。要深化服务业领域改革和创新，充分发挥服务业发展优势，挖掘服务业发展潜力，实施重大项目带动战略，全面推进服务业转型升级，进一步增强服务业特别是新型服务业在经济发展中的支撑作用。服务业范围广、行业门类多，都要抓好，今年重点要在以下几个方面有新突破。

以打造全国重要的云计算产业基地为目标，集中力量抓好云计算产业项目建设。在完成中国电信、中国联通数据中心一期项目并投入使用的基础上，尽快启动二期建设。促进中国移动数据中心项目全面开工建设，推动国家云应用检测中心、内蒙古盛乐软件园、大宗农畜产品电子交易平台、中国国际电子商务中心信息园落地建设。着力引进菜鸟网络、曙光、浪潮等一批有影响力的云计算相关行业龙头企业入驻，健全云计算产业链。依托云计算产业的发展，整合我市资源，建设我市云计算中心，大力发展政府公共服务、城市管理等云计算应用服务，推进智慧城市建设。推动和林盛乐云计算产业基地积极申报国家级高新技术特色产业化基地，积极争取我市被列入国家云计算创新服务试点城市、信息惠民国家示范城市。

以建设国内一流的旅游目的地城市为目标，统筹规划旅游景区布局，加快重点旅游景区改造、开发建设。完成中心城区大召区块、将军衙署周边、乌素图召、呼和浩特博物馆、成吉思汗大街的改造提升，巩固“国家历史文化名城”地位。认真谋划和推进旅游项目的深度开发，加快大青山草原马汇、清水河老牛湾国家地质公园、托克托黄河湿地及云中古郡、和林盛乐古城、武川大青山山地草原和森林、哈素海蒙元文化风景区的开发建设，推动旅游产业成为更大、更强的支柱产业。

以建设国际化的草原文化中心城市为目标，着力加快内蒙古(国际)文化产业新城等项目建设，推动大盛魁文化创意产业园申报国家级文化产业示范基地，打造文化产业核心区，促进文化产业集中集聚发展。继续深化文化体制改革，完成电影公司转制，培育壮大龙头文化企业，支持呼和浩特民族演艺集团开拓国际国内演出市场，完善文化人才培育引进和文化精品扶持奖励机制，加快出版发行、创意设计、新兴媒体、动漫游戏、文化艺术、艺术品交流等产业发展。

抓住我市被列入国家重要物流节点城市的机遇，优化调整布局，加快推进“两港、四区、两园、一带”物流基础设施网络建设。年内完成投资近10亿元的呼铁局沙良铁路大型综合物流港建设，改造白庙子铁路综合物流园区，以此形成立足全区、服务西北、面向全国、连接欧亚的铁路物流集散中心。完成城市东部、南部和西部沿绕城高速公路外侧4个大型仓储配送园区建设，逐步把煤炭、钢材、汽车等大宗物流全部迁出中心城区，完善城市配送服务体系，形成综合物流产业带。进一步做大做强和林大红城、托清两个大型煤炭专业物流园。依托新机场的建设，规划建设空港综合物流港，形成未来能够辐射周边城市的航空物流中心。

巩固提升我市在城市群中商业中心的地位，引进新型业态，进一步优化商业布局。巩固提升中山路等老商圈，重点打造新华东街商圈、东客站商圈、锡林南路商圈、滨河商圈、大商城等区域，增强大型商业综合体的新功能，充分发挥呼包鄂城际铁路、呼朔高速开通的优势，扩大对晋北、陕北等周边地区的辐射能力。继续办好中国民族商品交易会、呼和浩特昭君文化节、全国少数民族文化旅游艺术节、内蒙古绿色农畜产品博览会等会展活动，培育一批有影响力的会展品牌，促进会展业与旅游、商贸及相关产业协同发展，把我市建设成为北方极具民族地区特色、有影响力和吸引力的会展城市。

强力推动电子商务产业发展。抓住我市成为国家电子商务示范城市的重要契机，加快回民区金海电子商务基地、赛罕区毅德电子商务展示和交易区、盛乐现代服务业集聚区建设，着力抓好京东商城、苏宁云商集团等重大项目，积极支持内蒙商网、民族V购、宜齐网购等电子商务企业继续发展壮大，推动传统商贸企业和电子商务融合发展。

高度重视科技及职业教育产业发展。加快生产力促进中心等创新创业基地建设，发挥好大学科技园、留学人员创业园的作用，有效集聚科技创新资源要素，形成科技创新高地。进一步创新科研机构体制机制，支持内蒙古乳业技术研究院、内蒙古盛唐蒙医研究院加快发展，把科技创新、成果转化、科技服务产业结合起来，提升科技创新服务能力。面向产业发展和生活服务，整合提升我市职业教育资源，积极构建多元化的职业教育体系，促进职业教育产业加快发展。

加快养老和家政服务业的发展。积极引进高端知名家政企业，促进家庭服务企业规模化、品牌化和网络化发展。认真落实财政补贴、税收优惠、土地优惠政策，鼓励社会力量兴办养老机构、家政服务中心。培育3-5个家庭服务业龙头企业，构建100个社区家庭服务业网点。开工建设市级示范养老公寓、残疾人康复中心，开展公办养老机构改制试点，通过公建民营、民办公助等方式，完成30个以上社区老年人日间照料中心和15所互助养老幸福院的建设。

(四)加大工作力度，推动城市建设和管理再上新台阶

认真落实“改造提升中心城区，规划建设东部新区，打造北部生态观光带，建设南部现代产业园”的城市总体布局规划。

继续加大城市基础设施建设改造力度。老城区年内基本完成核心区道路改造，续建、新改造道路及地下管网12项，小街巷24条。改造老旧小区300个以上，全面实施小区供水、供热、供气服务延伸入户，切实解决提高居民供水及供热质量、环境综合治理、小区管理等问题。东部城区以实现近两年开工建设的桥梁、道路等基础设施项目互联互通为目标，初步构建起现代化的交通网络。加快推进市区分质分类供水工程，完成引黄入呼扩建工程，新建哈拉沁净水厂、金桥水厂，加大工业、园林绿化中水回用力度，做好高层建筑降水管控，建设节水型城市。城区新建设一座10万吨大型污水处理厂，对城区3个污水处理厂实施提标改造，切实减少污水排放。不断提升垃圾集中处理、分类处理水平，实施生活垃圾填埋气体开发利用工程，全面启动京城固废垃圾处理项目，规划建设扩大城市生活垃圾综合处理利用的新项目。高度重视房地产市场动态，及时调整政策及调控措施，确保我市房地产业平稳健康发展。

大力实施城市畅通工程。全面开工建设二环路改造城市快速路工程，年内努力实现60公里主线通车，从根本上解决城市中没有干线快速交通线路、交通拥堵、公共交通运行效率低的问题。对全市主要出城口进行综合整治和改造，提高出入车辆通行能力。全面实施创建国家公交都市示范城市计划，新建5个公交场站，新增公交车辆400台，推进线网优化工程，规划设置公交优先专用车道100公里，大幅度提高公共交通出行分担率。在全市主要街道、居民区、商业区增加布设公共自行车运营网点，投放公共自行车5000辆，倡导市民绿色出行。进一步优化完善城市智能交通系统、视频监控系统以及中心城区交通信息平台建设，提升全市交通组织管理水平和通行效率。

着力提高城市安居、宜居水平。全面推进47个城中村改造，新建续建回迁住房848万平方米。构建多层次、满足不同需求的住房供应保障体系，交付使用公租房5200套，新建公租房3000套，为更多中低收入家庭提供住房保障。完善400个老旧小区物业服务，提升小区居住品质。合理布局城市公共绿地，新建公园、游园6个，续建绿地8处，对103条道路绿化进行改造，形成若干城市园林小品，增加维护投入，提高园林养护水平。新建100个便民市场，改造城市边角地块30处，新增公厕85座，为市民营造舒适便利的生活环境。

切实提升城市环境空气质量。将今年确定为我市环境保护综合治理年。深入推进大青山前坡生态保护综合治理工程，开工建设塔利万亩生态公园和乌素图水库6000亩杏坞番红生态景区，续建5万亩森林公园，沿大青山前坡植树造林2万亩，提高绿化覆盖率。加大空气污染治理，全面完成电厂、热源厂脱硫脱硝工程，全年减少二氧化硫排放4906吨、氮氧化物排放4214吨。深入推进“气化呼和浩特”建设，对110座分散燃煤锅炉进行拆并整合和燃气化改造。加大城市扬尘治理，重点解决贮煤场、贮灰场、砂石场、建筑工地等扬尘污染问题。加强汽车尾气治理，全市公务用车率先推广使用机动车尾气净化装置，淘汰3000辆“黄标车”，机动车用油标准由国Ⅲ提高到国Ⅳ。努力把呼和浩特建成北方空气质量最好的城市之一。

全面推进城市精细化管理。创新城市管理模式，加大城市规划管理执行力度，严厉打击违法占地和违法建设行为，继续全面清理规范违法违规建设项目。提高道路机扫、洗扫覆盖率，合理配置保洁人员，实现城市主次干道“全天候”保洁和“无缝隙”管理。深入落实“门前三包”制度，强化部门单位的责任意识。将城市规划区内城中村纳入统一管理范围，消除管理盲区。加强户外广告标准化和规范化管理，实施好城市美化亮化工程，提升城市形象。加快提高数字化城管平台的应用水平，建立城市管理快速反应机制，创建安全、整洁、有序的城市环境。

(五)着力改善民生，全面提高公共服务水平

加快发展就业、社会保障、教育、医疗卫生等社会事业，切实解决好群众最关心最直接最现实的利益问题，努力为社会提供多样化服务，更好满足城乡居民需求。

积极创建创业型城市。全力组织实施创业就业工程，培育新的创业就业增长点，全面落实促进高校毕业生就业的实施意见，加快完成大学生创业就业服务中心建设，建立大学生创业进园区、就业服务进校区机制，力争使高校毕业生就业率达到90%以上。建设进城务工人员综合服务中心，稳定实现转移农村富余劳动力20万人。多措并举帮助6000名就业困难人员实现就业。

完善落实社会保障政策。认真落实企业退休人员基本养老金增长10%的政策，全面实施企业职工基本养老保险关系转移接续办法，完善被征地农民养老保险与城镇职工基本养老保险并轨办法，进一步做好新型农村社会养老保险、城镇居民社会养老保险制度合并实施工作。深入实施原国有集体企业下岗失业人员社会保险救助政策，出台解决原国有集体企业下岗失业断保人员养老保险接续办法。进一步完善城乡居民大病保险和救助制度，研究推动城镇医疗保险、新农合和城乡医疗救助制度在政策和管理上的衔接，推进制度整合。将新型农村合作医疗筹资标准提高到430元，大病补充保险最高支付限额提高到20万元，实际报销比例达到70%。调整城镇居民大额保险为大病补充保险，起付额由13万元降低到3万元，最高支付限额由8万元提高到10万元。加强低保与临时救助、城乡医疗救助等制度的衔接，完善社会救助体系。

坚持教育优先发展。调整优化学校布局，全市新建27所公办中小学，启动新一轮学前教育三年行动计划，新建改扩建公办幼儿园36所，严格执行新建住宅小区配建小学、幼儿园制度，引进国际先进教育理念，支持老牛基金会援建的儿童探索博物馆建设。优先重点发展民族教育，提高首府民族教育服务全区的能力。全面推进教育信息化工程三年行动计划，扩大优质教育资源覆盖面。改革中考招生考试制度，切实减轻课业负担，促进学生全面发展。全面做好呼和浩特职业学院新校区的配套建设，努力尽快升格为本科院校，提高我市高等教育办学水平。

深化医药卫生体制改革，加快公立医院改革，积极落实医师多点执业政策，促进优质医疗资源合理流动。优化医疗卫生资源布局，支持自治区新开工的内蒙古儿童医院、内蒙古医科大学第二附属医院等重点项目建设，完成市中蒙医院、市妇幼保健院等市级重点卫生项目建设，加强社区卫生服务中心标准化建设。加强自治区、市医疗机构与旗县医疗机构的联系，增强旗县农村和基层社区医疗机构服务能力。创新卫生人才引进、培养、使用制度，重视基层医疗卫生服务机构全科医生的培养。

加快公共服务设施网络建设。推进文化体制机制创新，鼓励社会力量、社会资本参与公共文化服务体系建设，推动公共文化服务社会化发展。年内新建6个小型剧场，推广区市两级及民间演艺团体驻场演出，全方位展示城市文化魅力。完善社区体育健身设施，年内新建6座小型体育综合场馆，广泛开展全民健身活动，增强市民身体素质。发挥好现有体育场馆作用，积极承办各类竞技体育赛事，促进体育产业健康发展。

扎实做好第三次经济普查工作，认真实施2011-2020呼和浩特市妇女儿童发展纲要，稳步推进其他各项社会事业。

(六)创新社会治理体制，营造和谐稳定发展环境

以解决事关人民群众切身利益与影响社会和谐的突出问题为突破口，创新社会治理体制，完善基层管理服务平台，加快和谐首府建设。

以社会服务管理网格化、社区建设规范化、村庄管理社区化为发展方向，加强基层基础工作。进一步完善社区网格化服务管理体系，加快市、旗县区两级信息网络服务平台建设，提高服务管理水平。规范和丰富社区网格化服务内容，积极探索农村网格化服务管理模式。加强社区用房和公益性服务设施建设，新建41个300平方米以上的社区服务站、4个5000平方米的精品示范社区服务站。深化乡镇、街道办事处的机构改革和职能转变，为居民提供更好更便利的服务。

创新有效预防和化解社会矛盾体制机制，及时有效地把各种社会矛盾化解在基层和萌芽状态，全力维护首府稳定。以解决问题为根本，严格实行市、旗县区两级领导干部信访工作责任制，加大对信访事项的解决力度，及时妥善解决征地拆迁、国企改制、工资拖欠等信访诉求。积极推进信访制度改革，采取网上受理和联合接访等方式，搭建多种形式的沟通对话平台，进一步畅通信访渠道。

高度重视安全生产，落实好地方政府、部门、企业的责任，突出抓好重点领域安全生产专项整治，保持安全生产形势稳定。高度重视食品药品质量安全问题，健全农产品生产、加工、流通各环节的质量安全和食品安全追溯体系，切实加大监管力度，确保广大人民群众“舌尖上的安全”。推动“中国呼和浩特国家级乳肉制品检测中心”全面启用。大力整顿和规范市场秩序，全面加强产品质量安全监管，保护消费者合法权益。加强防灾减灾体系建设，全面改造提升消防基础设施水平，提高灾害防御能力。健全突发事件工作机制，提高城乡应急服务管理和处置水平。

加强社会治安防控，严厉打击暴力犯罪和“两抢一盗”，推进平安首府建设。加强互联网管控，依法打击利用互联网进行渗透犯罪活动，营造积极健康的网络环境。全面启动法治呼和浩特建设，深入推进“六五”普法工作。深入贯彻党的民族宗教政策，广泛开展民族团结进步宣传和创建活动。坚持走军民融合式发展道路，全力支持国防和军队现代化建设，加强国防教育和民兵预备役建设，做好拥军优属工作，争创全国双拥模范城八连冠。

坚持以社会主义核心价值观引领社会进步。深入开展理想信念教育和爱国主义、集体主义、社会主义教育，广泛开展道德实践活动。全面推进全国文明城市创建，开展文明社区、文明村镇、文明单位、文明家庭创建活动，提升市民文明素质。更加注重内涵建设和服务群众，广泛开展道德模范评选活动，大力弘扬真善美，传递正能量。凝炼能够激发首府人民创业热情、展现首府蓬勃向上风貌的文化符号和城市精神，提升市民对城市的认同感、归属感和自豪感。

(七)全面深化改革，加强政府自身建设

全面落实党的十八届三中全会精神，准确把握深化改革的方向和重点，坚持问题导向的改革意识和改革思路，全面深入推进各项改革工作，用改革的思路和办法破解发展难题，激发市场活力，增强经济发展的内生动力，既要主动而为，又要搞好同中央及自治区的衔接。

坚持从实际出发，优化政府组成部门及办事机构设置，9月底前完成市、县两级政府机构改革，提升政府工作效率。全面清理妨碍公平竞争的有关规定和做法，深化行政审批制度改革，今年将原市级264项行政审批及许可事项减少到92项，实施中还要继续加大减少和下放力度，进一步向市场放权、向基层放权，为企业松绑，为群众提供便利。

深化财税体制改革，改进预算管理，完善部门预算编制办法和执行管理办法，形成规范透明的预算管理制度。合理控制政府债务规模，规范偿债基金运行，正确处理好举债与发展的关系。继续加大政策扶持，优化环境，着力吸引国内外各类金融保险基金证券等企业来我市设立分支机构。鼓励各类资本参与地方金融机构增资扩股，发起设立民营银行，加快村镇银行、资金互助社等新型金融机构组建，促进金融业健康快速发展。

围绕使市场在资源配置中起决定性作用和更好发挥政府作用，鼓励支持非公有制经济发展。废除对非公有制经济的不合理规定，实行统一的市场准入制度。认真落实鼓励民间投资、支持非公有制经济发展的政策措施，鼓励民间资本进入重点产业、基础设施建设和公共服务领域，引导中小企业、民营企业搞好配套协作。成立呼和浩特市中小企业担保有限责任公司，设立中小企业发展基金，强化中小企业融资担保、政策性贴息及发展成果奖励，破解中小企业融资难问题，充分释放非公有制经济发展活力和创造力。

坚持把依法行政贯穿于政府工作全过程，自觉接受人大及其常委会的法律监督，认真落实其各项决议、决定并定期报告工作。主动接受人民政协的民主监督及社会各界的监督，支持政协参政议政，落实好年度协商计划。加快法治政府建设，最大限度减少体制和制度漏洞。建立健全重大问题集体决策、专家咨询论证、社会公示、听证和决策跟踪反馈机制，促进科学民主依法决策。加大政务公开力度，畅通政府与公众互动渠道，让人民群众更有效地监督政府，促进政府工作做的更好。

深入推进惩治和预防腐败体系建设，强化对权力运行的制约和监督。认真落实党风廉政建设责任制，严格执行廉洁从政各项规定和政府各项工作规则，坚持以制度管权、管事、管人。加强政府层级监督，强化对重要领域、关键岗位的行政监察和审计监督，严肃查处违纪违法案件。充分发挥公共资源交易中心的职能和作用，规范公共资源交易行为，从源头上杜绝工程建设等领域腐败。

认真开展以“为民务实清廉”为主题的党的群众路线教育实践活动，按照“照镜子、正衣冠、洗洗澡、治治病”的总要求，深入开展批评与自我批评，着力解决政府组成部门及政府工作人员在“四风”方面存在的突出问题。继续巩固全面清理治理超标准办公用房和超标超编公务用车成果，进一步压减“三公”经费和一般性支出，把有限的资金和资源更多更好地用于发展经济和改善民生。加快转变政府职能，改进政府工作作风，教育引导全体政府工作人员克己奉公、勤政廉洁，真正把心思用在干事创业上，始终保持昂扬向上、奋发有为的精神状态，以踏石留印、抓铁有痕的工作劲头，推动首府各项事业再上新台阶。

(八)全面启动迎接自治区成立70周年各项工作

一是把迎庆项目建设作为一项重大政治任务，按照填补空白、提高水平、具有较强示范带动作用，全面提升服务全市人民乃至服务全区的能力，确保于2016年或2017年夏季前竣工的基本原则，市政府要及早谋划一批重大工程和项目。

二是加快推进自治区及我市已确定并启动的迎庆重点项目建设。今年要力争呼张高铁我市段全线开工;机场迁建、城铁项目获批;确保二环快速路全线开工、力争年内主线通车。

三是加大工作力度，确保我市今年成为全国文明城市创建提名资格城市，到2017年相继进入国家环保模范城市、国家园林城市、国家卫生城市、全国文明城市行列。

四是加强与自治区的沟通对接，统筹协调各项迎庆工作。

各位代表，新一轮深化改革的征程已经开启，推动创新发展的使命光荣而艰巨。让我们在自治区党委、政府和市委领导下，团结带领全市各族人民，凝心聚力，攻坚克难，扎实工作，用我们的勤劳和智慧，共同开创呼和浩特更加美好的未来!
